# ATP exposure stimulates glutathione efflux as a necessary switch for NLRP3 inflammasome activation

**DOI:** 10.1016/j.redox.2021.101930

**Published:** 2021-03-10

**Authors:** Tianli Zhang, Hiroyasu Tsutsuki, Waliul Islam, Katsuhiko Ono, Kohsuke Takeda, Takaaki Akaike, Tomohiro Sawa

**Affiliations:** a Department of Microbiology, Graduate School of Medical Sciences, Kumamoto University, 1-1-1 Honjo, Chuo-ku, Kumamoto, 860-8556, Japan; b Department of Cell Regulation, Graduate School of Biomedical Sciences, Nagasaki University, 1-14 Bunkyo-machi, Nagasaki, 852-8521, Japan; c Department of Environmental Medicine and Molecular Toxicology, Tohoku University Graduate School of Medicine, 2-1, Seiryo-machi, Aoba-ku, Sendai, Miyagi, 980-8575, Japan

**Keywords:** NLRP3 inflammasome, Glutathione, ATP, GSH efflux, Reactive oxygen species, Redox regulation

## Abstract

The NLRP3 inflammasome is a multiprotein complex responsible for the maturation of precursor forms of interleukin (IL)-1β and IL-18 into active proinflammatory cytokines. Increasing evidence suggests that modulation of redox homeostasis contributes to the activation of the NLRP3 inflammasome. However, specific mechanistic details remain unclear. We demonstrate here that ATP exposure evoked a sharp decrease in glutathione (GSH) levels in macrophages, which led to NLRP3 inflammasome activation. We detected an increase in GSH levels in culture supernatants that was comparable to the GSH decrease in macrophages, which suggests that exposure to ATP stimulated GSH efflux. Exogenous addition of P2X7 receptor antagonist, GSH, or the oxidized form GSSG attenuated this efflux. Also, exogenous GSH or GSSG strongly inhibited NLRP3 inflammasome activation *in vitro* and *in vivo*. These data suggest that GSH efflux controls NLRP3 inflammasome activation, which may lead to development of novel therapeutic strategies for NLRP3 inflammasome-associated disorders.

## Highlights

• ATP stimulates rapid glutathione (GSH) efflux via the P2X7 receptor.
• GSH efflux is an upstream event for NLRP3 inflammasome complex assembly.
• Exogenous GSH weakens ATP-caused GSH efflux and NLRP3 inflammasome activation.
• GSH or GSSG suppressed interleukin-1β production in an inflammatory mouse model.

## Graphical abstract

**Figure:**
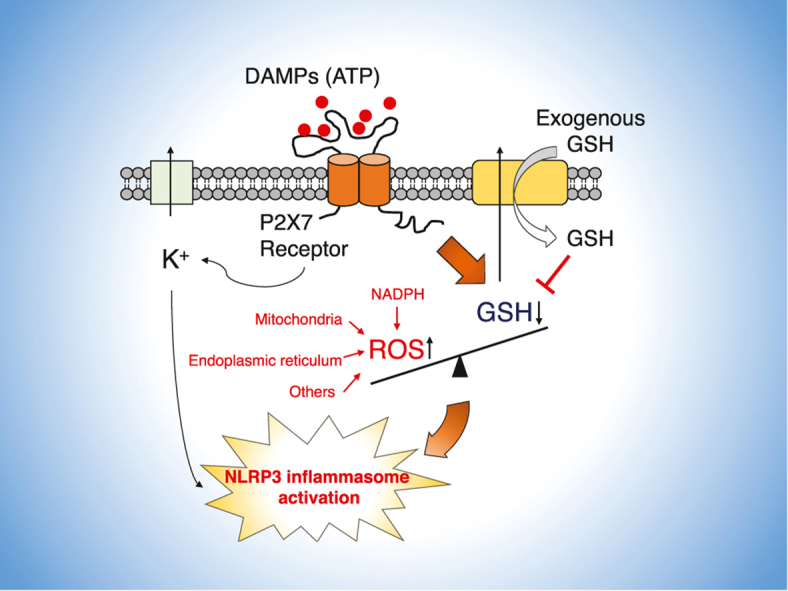

## Abbreviations

ASC: apoptosis-associated speck-like protein containing a CARD
BMDMs: bone marrow-derived macrophages
BSO: l-buthionine-(*S*,*R*)-sulfoximine
CBX: carbenoxolone
CysSH: cysteine
Cys-Gly: cysteinylglycine
DAMPs: damage-associated molecular patterns
GSDMD: gasdermin D
GSH: glutathione
GSH-EE: glutathione reduced ethyl ester
γ-Glu-Cys: γ-glutamylcysteine
GSSH: glutathione persulfide
GSSSH: glutathione polysulfide
GSSG: oxidized glutathione
IL: interleukin
isotope-labeled GSH: GSH-(*glycine*-^13^C_2_,^15^N)
K^+^: potassium
LC-MS/MS: liquid chromatography-tandem mass spectrometry
LPS: lipopolysaccharide
mROS: mitochondria-derived ROS
Nek7: NIMA-related kinase 7
NLRP3: nod-like receptor protein 3
NOX: NADPH oxidase
poly(dA:dT): poly(deoxyadenylic-deoxythymidylic) acid
ROS: reactive oxygen species
*S.* Typhimurium LT2: *salmonella enterica* serovar Typhimurium strain LT2
TNF-α: tumor necrosis factor-α
TRX: thioredoxin
TXNIP: thioredoxin-interacting protein
KCl: potassium chloride.

## 1 Introduction

Inflammasomes are intracellular multiprotein complexes that are involved in both host defense and inflammatory responses via the maturation of proinflammatory cytokines including pro-interleukin (IL)-1β and pro-IL-18, as well as the initiation of pyroptotic cell death [1]. Among the various inflammasomes, the NLRP3 inflammasome has been the most extensively studied and consists of a pattern recognition receptor (NLRP3), apoptosis-associated speck-like protein containing a CARD (ASC), pro-caspase-1, and a recently identified component NIMA-related kinase 7 (Nek7) [2]. Dysregulation of the NLRP3 inflammasome has been associated with the pathogenesis of various inflammatory diseases [3]. NLRP3 inflammasome activation occurs in two distinct steps—priming and activation [4]. During the priming step, transcriptional upregulation of NLRP3 inflammasome components including NLRP3, pro-caspase-1, and pro-IL-1β can be induced by the recognition of various pathogen-associated molecular patterns such as lipopolysaccharide (LPS), which leads to nuclear factor-κB signaling activation [4]. Compared with other inflammasomes, the NLRP3 inflammasome is unique in that its activation step is induced by diverse damage-associated molecular patterns (DAMPs), such as extracellular ATP; nigericin, a pore-forming ionophore; and crystalline particles [4]. Potassium (K^+^) efflux in this step is believed to be a common event upstream of NLRP3 inflammasome activation [5]. This K^+^ efflux drives interactions between Nek7 and NLRP3, which results in assembly of the NLRP3 inflammasome complex and processing of other cytokines [2].

Generation of reactive oxygen species (ROS) was proposed as an additional signal for NLRP3 inflammasome activation, although this proposal was controversial [6]. NADPH oxidase (NOX)-derived ROS were originally thought to be necessary for NLRP3 inflammasome activation [7]. Ma et al. found that NOX2 knockout disturbed NLRP3 inflammasome activation in the cerebral cortex but not in the umbilical vein endothelium, which suggests a tissue-specific role of NOX2-mediated ROS in NLRP3 inflammasome activation [8]. In addition, several studies demonstrated that the effects of genetic or pharmacological inhibitors of NOX on NLRP3 inflammasome activation were ambiguous [[9], [10], [11]]. Ives et al. reported that ROS generated by xanthine oxidase promoted IL-1β secretion on NLRP3 inflammasome activation, whereas pharmacological inhibition of xanthine oxidase did not completely inhibit NLRP3 inflammasome activation, especially in the model of the ATP-activated NLRP3 inflammasome [12]. Increasing evidence has suggested that, in addition to ROS-generating enzymes, mitochondria-derived ROS (mROS) contribute, via the mitochondrial respiratory chain, to NLRP3 inflammasome activation in response to myriad activators [13]. Other studies, however, argued that mROS are not essential for NLRP3 inflammasome activation [5,14]. Despite the lack of understanding of the detailed mechanisms of how ROS from different sources regulate NLRP3 inflammasome activation, the aforementioned models suggest that a broad association exists between ROS and the NLRP3 inflammasome.

Glutathione (γ-glutamyl-cysteinyl-glycine, GSH) is a tripeptide formed by glutamate, cysteine, and glycine and is the most abundant low-molecular-weight thiol in mammalian cells. In general, GSH function contributes significantly to the maintenance of the redox milieu in cells via both direct and indirect ROS scavenging [15]. GSH also participates in post-translational modification of cysteine residue-dependent proteins, called S-glutathionylation [16]. Recent advances in proteomic analysis of NLRP3 inflammasome components revealed that the occurrence of S-glutathionylation is associated with NLRP3 inflammasome activation [[17], [18], [19]]. In light of these findings, we hypothesized that GSH together with cellular ROS may be involved in the regulation of NLRP3 inflammasome activation.

In this study, we analyzed both the dynamic changes in intracellular GSH levels and the corresponding generation of ROS after NLRP3 inflammasome activation in macrophages. We also investigated the biological effects of GSH on NLRP3 inflammasome activation both *in vitro* and *in vivo*. Our data demonstrated that rapid GSH efflux occurred in the proximal upstream region of NLRP3 inflammasome activation, which led to concomitant generation of ROS. GSH efflux specifically responded to ATP stimulation initiated via P2X7 receptor-mediated signaling. GSH efflux stopped after exogenous addition of GSH, or its oxidized form GSSG, and this effect was associated with suppression of IL-1β release. Our findings provide insight into redox regulatory mechanisms of NLRP3 inflammasome activation and hence identify potential therapeutic targets in NLRP3 inflammasome-associated disorders.

## 2 Materials and methods

### 2.1 Reagents

Isoflurane inhalation solution was obtained from Pfizer Japan Inc (Tokyo, Japan). Red Blood Cell Lysing Buffer (R7757), LPS from *Escherichia coli* O55:B5 (L2880), LPS from *E. coli* O111:B4 (L2630), ATP (A6419), 2ʹ,7ʹ-dichlorodihydrofluorescein diacetate (DCF-DA) (D6883), DIDS (D3514), CBX disodium salt (C4790), Gap19 trifluoroacetate salt (SML1426), GSH-EE (G1404), Cys-Gly (C0166), γ-Glu-Cys (G0903), isotope-labeled GSH (638620), GSH (G6529), and GSSG (G4376) were purchased from Sigma-Aldrich (St. Louis, MO, USA). Recombinant mouse M-CSF (576404), collagen type I (from calf skin, 150026) and PolyFect Transfection Reagent (301107) were obtained from BioLegend (San Diego, CA, USA), MP Biomedicals (Santa Ana, CA, USA), and QIAGEN (Hilden, Germany) respectively. Bis(sulfosuccinimidyl)suberate disodium salt (BS3) (B574) was purchased from Dojindo (Kumamoto, Japan). Nigericin sodium salt (145-07263), AMP (492375), ADP (019-25091), adenosine (015-24591), A-804598 (014-25301), BSO (021-14121), PAH (010-12191), BFA (022-15991), rifamycin SV sodium salt (590-29991), gentamicin sulfate (075-06451), phosphatase inhibitor cocktail solution I (167-24381), protease inhibitor cocktail I (165-26201), BCA Protein Assay Kit (297-73101), anti-DYKDDDDK tag antibody (014-22383), and anti-β-actin antibody (281-98721) were obtained from FUJIFILM Wako Pure Chemical Corporation (Osaka, Japan). FluxOR II Green Potassium Ion Channel Assay (F20015), recombinant protein G agarose (15920010), and Lipofectamine 2000 transfection reagent (11668027) were purchased from Thermo Fisher Scientific (Waltham, MA, USA). Glibenclamide (G0382), phloretin (P1966), *N*-ethylmaleimide (E0136), 4ʹ-hydroxy-3ʹ-methoxyacetophenone (apocynin) (H0261), and monobromobimane (MBB) (B4220) were purchased from Tokyo Chemical Industry Company, Ltd (Tokyo, Japan). Guanosine-5ʹ-triphosphate disodium salt (GTP) (17450-61), l-cysteine (10309-12), KCl (28514-75), and penicillin-streptomycin mixed solution (26253-84) were purchased from Nacalai Tesque (Kyoto, Japan). Poly(dA:dT) (tlrl-patn-1), H-Glu(Gly-Gly–OH)–OH (4000347), and Ko143 (3241) were obtained from InvivoGen (San Diego, CA, USA), Bachem (Torrance, CA, USA), and Tocris Bioscience (Bristol, UK), respectively. MK571 (70720) was purchased from Cayman Chemical Company (Ann Arbor, MI, USA). Anti-NLRP3 (D4D8T) antibody (#15101), anti-ASC (D2W8U) antibody (#67824), anti-rabbit IgG HRP-linked antibody (#7074), and anti-mouse IgG HRP-linked antibody (#7076) were purchased from Cell Signaling (Danvers, MA, USA). Anti-caspase-1 (p20) antibody (AG-20B-0042-C100) was purchased from AdipoGen (San Diego, CA, USA). Anti-Nek7 antibody (ab133514) and anti-gasdermin D (GSDMD) antibody (ab209845) were obtained from Abcam (Cambridge, UK). Anti-GSH antibody (101-A-100) was purchased from Virogen Corporation (Watertown, MA, USA).

### 2.2 Animal study

ICR mice (male, 8 weeks old) and C57BL/6 N mice (male, 10 weeks old) were purchased from Japan SLC Inc. (Shizuoka, Japan) and maintained at the Center for Animal Resources and Development, Kumamoto University. All procedures were carried out according to the Kumamoto University Ethics Review Committee for Animal Experimentation (Experiment number A30–064 and A2020-084) and were performed to minimize the animals’ suffering, as well as the number of animals used. A cytokine hyperproduction mouse model was used to study the effects of GSH on *in vivo* NLRP3 inflammasome-mediated IL-1β production [20]. ICR mice were randomly divided into five groups of five mice to receive different treatments: control, LPS, LPS/ATP, LPS/ATP plus GSH, and LPS/ATP plus GSSG. After measurement of the body weight of the ICR mice, the mice (average body weight about 35 g) were injected intraperitoneally with either phosphate-buffered saline (PBS) (0.1 mL, solvent control) or LPS [from *E. coli* O111:B4, 2 μg/kg body weight (0.1 mL of 700 ng/mL)]. After 90 min, the mice received intraperitoneal injections of physiological saline (0.35 mL, solvent control), ATP [50 μmol/kg body weight (0.35 mL of 5 mM)], or ATP plus GSH or GSSG (same concentration as that of ATP). One hour later, whole blood samples were collected from each group of mice, with serum samples obtained via centrifugation. Serum IL-1β and TNF-α levels were quantified as described below.

### 2.3 Cell preparation and culture

Cells of the murine macrophage cell line J774.1 (RCB0434) were purchased from Cell Engineering Division/RIKEN BioResource Research Center (Tsukuba, Japan). Cells of the human embryonic kidney cell line HEK293FT were a gift from Prof. Hideshi Ihara (Graduate School of Science, Osaka Prefecture University). All cells were cultured in Dulbecco's Modified Eagle's Medium (DMEM) (FUJIFILM Wako Pure Chemical Corporation) supplemented with 10% heat-inactivated fetal bovine serum (MP Biomedicals) and 1% penicillin-streptomycin (Nacalai Tesque) in a 5% CO_2_ humidified incubator at 37 °C. BMDMs were isolated from C57BL/6 N mice [21] as follows: mice were anesthetized with isoflurane and euthanized via cervical dislocation. Bone marrow cells were then harvested from the femurs and tibias via repeated flushing. After centrifugation, erythrocytes were removed by using Red Blood Cell Lysing Buffer for 2 min at room temperature. The remaining cells were cultured in complete DMEM containing 50 ng/mL recombinant mouse M-CSF for 1 week to form BMDMs.

### 2.4 Inflammasome activation

BMDMs (5 × 10^5^ cells/mL) and J774.1 cells (5 × 10^5^ cells/mL) were seeded in 96-, 24- or 6-well plates. After overnight incubation, cells were either primed with LPS (100 ng/mL) for 5 h or were not primed. Cells were then stimulated for 1 h with inflammasome activators or other stimuli: ATP (5 mM), nigericin (10 μM), AMP (5 mM), GTP (5 mM), ADP (5 mM), or adenosine (5 mM). GSH or its derivatives were added simultaneously at the indicated concentrations when the cells were treated with ATP. To activate the AIM2 inflammasome, poly(dA:dT) (1 μg/mL) was transfected into LPS-primed BMDMs or J774.1 cells with Lipofectamine 2000 transfection reagent for 6 h. For NLRC4 inflammasome activation, complete DMEM was replaced by antibiotic-free DMDM before LPS priming. *S.* Typhimurium LT2 (Laboratory strain) was diluted in PBS to achieve a multiplicity of infection of 1, after which it was added to LPS-primed BMDMs or J774.1 cells. Cells were incubated for 30 min, and then they were incubated in 100 μg/mL gentamicin-containing DMDM for 2 h, followed by additional incubation with 20 μg/mL gentamicin-containing DMEM until 20 h after infection. To remove intracellular GSH, J774.1 cells were pre-incubated with BSO (200 μM) for 16 h. After confirmation of the intracellular GSH level, cells were used for studies of NLRP3 inflammasome activation by the same method. Other inhibitors or blockers were added at the indicated concentrations to cells 1 h before ATP stimulation.

### 2.5 Cell death assay

Cytoprotective effect of GSH was determined by trypan blue dye exclusion staining. In brief, J774.1 cells were pre-seeded in 96-well plate at a density of 5 × 10^5^ cells/mL. On the following day, cells were primed with LPS (100 ng/mL) for 5 h, followed by stimulation with ATP (5 mM) in the absence or presence of GSH (5 mM) or GSSG (5 mM). After incubation for further 6 h, cells were detached using trypsin-EDTA. Subsequently, cell suspension was mixed with 0.4% trypan blue stain solution (Thermo Fisher Scientific) at a ratio of 1 : 1. Ten microliter of mixture was subjected to cell counting chamber slides (Thermo Fisher Scientific), and cell viability was calculated by Countess Automated Cell Counter (Thermo Fisher Scientific).

### 2.6 GSH metabolomic analyses

GSH metabolomic analyses were performed by using liquid chromatography-electrospray ionization-mass spectrometry with the Agilent 6460 Triple Quadrupole LC-MS system (Agilent Technologies, Santa Clara, CA, USA). To quantify intracellular GSH and its derivatives, we used the MBB derivatization method as reported previously [22,23]. Treated cells were washed with PBS once and harvested in methanol containing 5 mM MBB. Cell samples were then homogenized by using Bioruptor UCD-250 (Tosho Electronic, Tokyo, Japan) for 2 min, followed by an incubation at 37 °C. Thirty minutes later, supernatants were separated via centrifugation and were diluted with 0.1% formic acid containing known amounts of isotope-labeled standards. Mixtures were then subjected to LC-MS/MS metabolomic analysis. Precipitates were resuspended with 1% SDS in PBS and were then homogenized with a Bioruptor for 10 min, after which samples were used for quantifying protein concentrations with the BCA Protein Assay Kit (FUJIFILM Wako Pure Chemical Corporation). LC-MS/MS conditions were as follows: column, YMC-Triart C18 Plus column (2.1 × 50 mm) (YMC Co. Ltd., Kyoto, Japan); column temperature, 45 °C; injection volume, 10 μL; mobile phases: A, 0.1% formic acid, and B, acetonitrile; gradient (B concentration), 0 min–1%, 10 min–80%, 10.5 min–1%, 15 min–1%; and flow rate, 0.2 mL/min. The general conditions for ESI-MS were nebulizer gas, nitrogen, delivered at 50 psi; nebulizer gas temperature, 250 °C; capillary voltage, 3500 V; collision gas, and G1 grade, nitrogen (Taiyo Nippon Sanso Corporation, Tokyo, Japan). Table S1 provides details of the multiple reaction monitoring (MRM) parameters that we used in this study.

### 2.7 GSH efflux assay

In an attempt to analyze extracellular GSH levels, BMDMs or J774.1 cells were seeded in 24-well plates, both at densities of 5 × 10^5^ cells/mL. After overnight incubation, cells were primed with LPS (100 ng/mL) for 5 h before being washed with l-methionine- and l-cystine-free DMEM (D0422, Sigma-Aldrich) supplemented with 2 mM l-glutamine, 10% fetal bovine serum, and 1% penicillin-streptomycin. Then, ATP (5 mM)-containing complete l-methionine- and l-cystine-free DMEM was added to the cells to prevent reactions between culture medium components and GSH or its metabolites. After incubation, culture supernatants and cells were harvested and analyzed with LC-MS/MS.

### 2.8 Transfection of HEK293FT cells

HEK293FT cells were seeded at a density of 1 × 10^5^ cells/mL in a 24-well plate coated with collagen type I (10 μM). After replacement of medium with antibiotic-free DMEM, cells were transiently transfected with vectors expressing flag-P2X7 (wild type), Δc-P2X7 (mutant) in a pcDNA3 backbone, or empty pcDNA3 vector (control), as reported previously [24]), by using PolyFect transfection reagent according to the manufacturer's instructions. Forty-eight hours later, these transfected cells were used for GSH efflux assays.

### 2.9 siRNA knockdown

To knockdown GSDMD in J774.1 cells, cells were pre-seeded in 24-well plate at a density of 1 × 10^5^ cells/mL and cultured in antibiotics-free DMEM. After incubation for overnight, si-GSDMD (100 nM) or its-related negative control (si-NC) (100 nM) were transfected into J774.1 cells using Lipofectamine RNAiMAX transfection reagent (Thermo Fisher Scientific) according to the manufacturer's instructions. After transfection for 24 h, siRNAs (100 nM) were added again into cells. Forty-eight hour later, some of these cells were used for the confirmation of GSDMD expression via immunoblotting, and the other part was used for studies of ATP-induced GSH efflux. The siRNA was synthesized by Sigma-Aldrich Japan (Tokyo, Japan), and the sequences of si-GSDMD as follow:

sense: 5ʹ-GGUGAACAUCGGAAAGAUU-3ʹ
anti-sense: 5ʹ-AAUCUUUCCGAUGUUCACC-3ʹ.

### 2.10 Measurement of ROS generation

DCF-DA was used to detect ROS generation in response to various inflammasome activators. Activator-treated BMDMs or J774.1 cells were washed with modified Ringer's buffer (110 mM NaCl, 5 mM KCl, 2 mM CaCl_2_, 44 mM NaHCO_3_, 1 mM MgSO_4_, 1 mM NaH_2_PO_4_, and 5.5 mM glucose), followed by incubation with 5 μM DCF-DA-containing modified Ringer's buffer at 37 °C for 15 min in darkness. Fifteen minutes later, images were obtained with a BZ-X700 fluorescence microscope (Keyence Corporation, Osaka, Japan). A BZ-X analyzer (Keyence Corporation) was used for additional image processing and quantification.

### 2.11 Quantification of cytokine production

Levels of cytokines, including IL-1β and TNF-α, both in culture supernatants and in serum, were measured by using the mouse IL-1β/IL-1F2 Quantikine ELISA Kit (MLB00C; R&D Systems, Inc., Minneapolis, MN, USA) and mouse TNF-α Quantikine ELISA Kit (MTA00B; R&D Systems, Inc.), respectively, according to the manufacturer's instructions. Absorbance at 490 nm was then measured with an iMark Microplate Reader (Bio-Rad Laboratories, Hercules, CA, USA).

### 2.12 Determination of intracellular K^+^ concentrations

Intracellular K^+^ concentrations were determined with a FluxOR II Green Potassium Ion Channel Assay Kit according to the manufacturer's instructions. J774.1 cells were plated in a Corning 96-well black polystyrene microplate. On the day of the experiment, J774.1 cells were first primed with LPS (100 ng/mL) for 5 h before being treated with ATP (5 mM), either in the absence or presence of GSH (5 mM), GSSG (5 mM), or KCl (10 mM) for 30 min. After cells were washed once with DMEM medium, they were loaded with thallium-containing buffer for 1 h in darkness. A high K^+^ stimulus buffer was then added without removing the original buffer. After a 15-min incubation at 20 °C, fluorescent signals were detected by using a Tecan Infinite 200 Pro plate reader (LabX, Midland, ON, Canada), with excitation/emission wavelengths of 485/535 nm, respectively. All reagents in this assay were provided by the FluxOR II Green Potassium Ion Channel Assay Kit.

### 2.13 Co-immunoprecipitation

A pull-down buffer (50 mM Hepes-NaOH pH 7.5, 15 mM NaCl, 1 mM EGTA pH 8, 150 mM MgCl_2_, 10% glycerol, 1% Triton X-100, phosphatase inhibitor cocktail I, and protease inhibitor cocktail I) was added to J774.1 cells treated under certain conditions, and these cells were sonicated with a Bioruptor UCD-250 (Tosho Electric, Tokyo, Japan). After centrifugation at 14,000 *g* at 4 °C for 5 min, supernatants were incubated with recombinant protein G agarose at 4 °C to remove nonspecific binding proteins. One hour later, these supernatants were collected via centrifugation at 14,000 *g* at 4 °C for 5 min and were incubated overnight with either anti-NLRP3 antibody or anti-caspase-1 (p20) antibodies. The next day, the samples were incubated with recombinant protein G agarose at 4 °C for 2 h. After the samples were washed twice with a pull-down buffer, the protein complexes were analyzed via Western blotting.

### 2.14 ASC oligomerization

BMDMs were washed with cold PBS once and homogenized with a lysis buffer (20 mM Hepes-NaOH pH 7.5, 150 mM KCl, 1% NP-40, phosphatase inhibitor cocktail I, and protease inhibitor cocktail I). NP-40-soluble fractions (supernatants) and -insoluble fractions (pellets) were separated by centrifugation at 3300*g* at 4 °C for 10 min. NP-40-insoluble fractions were then resuspended for 45 min in a BS3 (4 mM)-containing CHAPS buffer (20 mM Hepes-NaOH pH 7.5, 5 mM MgCl_2_, 0.5 mM EGTA, and 0.1% CHAPS) to crosslink ASC monomers. Finally, samples were dissolved in Laemmli SDS sample buffer (62.5 mM Tris-HCl pH 6.8, 2% SDS, 6% glycerol, 2.5% 2-mercaptoethanol, and 0.005% bromophenol blue).

### 2.15 Western blotting

Cell samples were lysed in Laemmli SDS sample buffer before being boiled for 5 min at 98 °C. These protein samples were then subjected to SDS-PAGE. Subsequently, separated proteins were transferred to PVDF membranes (Immobilon-P; EMD Millipore, Darmstadt, Germany). Membrane blocking was performed with 5% skim milk in Tris-buffered saline (TBS) containing 0.1% Tween 20 (TBS-T) (20 mM Tris pH 7.5, 137 mM NaCl, and 0.1% Tween 20). After a 1-h incubation, membranes were incubated overnight at 4 °C with the primary antibodies against NLRP3, caspase-1, Nek7, ASC, GSDMD and FLAG. After washing the membranes with TBS-T, secondary antibodies including anti-mouse or anti-rabbit IgG HRP-linked antibody were used in additional reactions. Protein bands on the membranes were detected with Immobilon Western Chemiluminescent HRP Substrate (Merck Millipore, Burlington, MA, USA) and the luminescent image analyzer ChemiDoc XRS system (Bio-Rad Laboratories).

### 2.16 Statistical analysis

Each experiment in this study was performed at least three times independently. The data are presented as the mean ± standard deviation (SD). Statistical analyses were performed by using Student's *t*-test or one-way ANOVA followed by Tukey's multiple comparison test with GraphPad Prism 7.0 (GraphPad Software, La Jolla, CA, USA). A p-value less than 0.05 was considered to be statistically significant.

## 3 Results

### 3.1 GSH is depleted during NLRP3 inflammasome activation in response to exposure to ATP

**Fig. 1:**
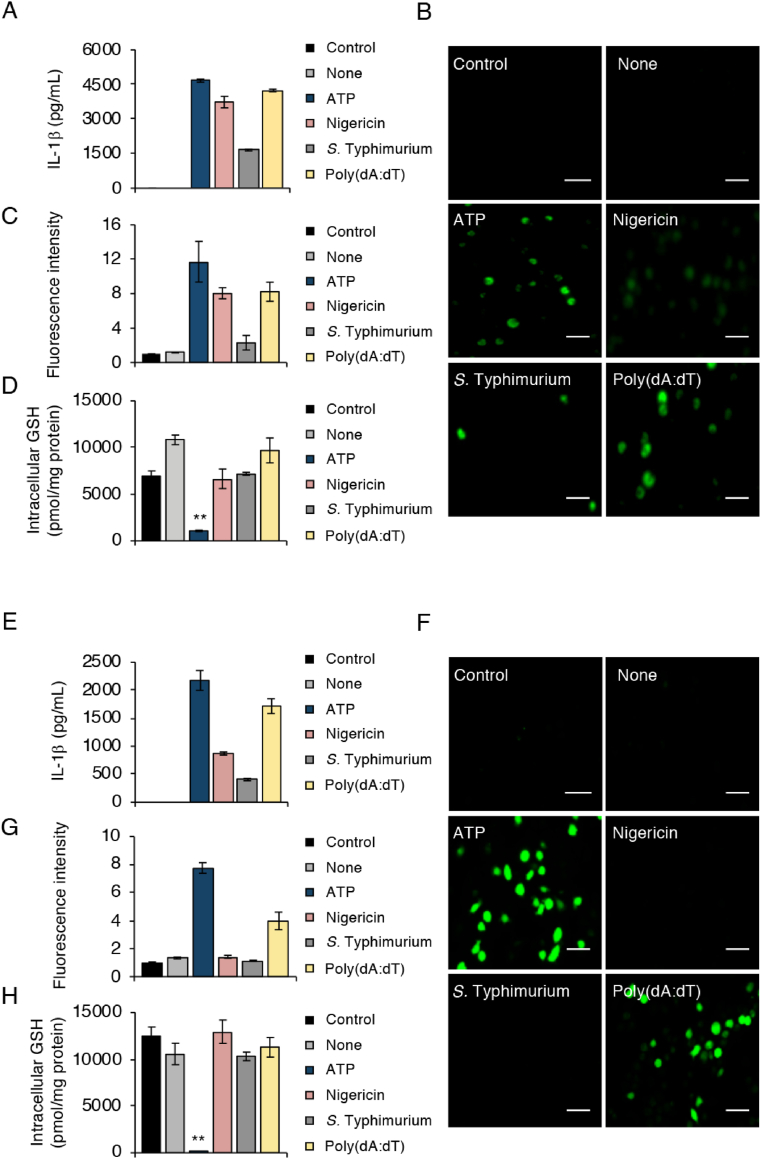
**ATP treatment induces GSH depletion during NLRP3 inflammasome activation.** (A–D) BMDMs were primed with LPS (100 ng/mL) for 5 h and then treated with 5 mM ATP for 1 h, 10 μM nigericin for 1 h, *S.* Typhimurium LT2 at a multiplicity of infection 1 for 20 h, or 500 ng/mL poly(dA:dT) for 6 h. (A) IL-1β levels in culture supernatants were quantified via ELISA; (B) ROS generation was detected by using DCF-DA. (C) Quantitative data for results in (B). (D) Intracellular GSH levels were measured by means of LC-MS/MS. (E–H) LPS-primed J774.1 cells were stimulated under the same conditions as those for BMDMs in (A–D). (E) IL-1β production, (F) ROS generation, and (H) intracellular GSH levels were analyzed with the same methods as those used in (A–D). (G) Quantitative data for results in (F). Scale bars: 50 μm. Controls were cells not treated with LPS or activators. Data represent means ± standard deviation (SD) (n = 3). **p < 0.01.

As assessed by IL-1β production in LPS-primed bone marrow-derived macrophages (BMDMs), we successfully activated the NLRP3 inflammasome against ATP and nigericin after 1 h stimulation (Fig. 1A). Longer stimulation period was required to fully activate the NLRC4 inflammasome against *Salmonella enterica* serovar Typhimurium strain LT2 (*S.* Typhimurium LT2) (20 h), and the AIM2 inflammasome against poly(deoxyadenylic-deoxythymidylic) acid [poly(dA:dT)] (6 h) for equivalent IL-1β production (Fig. 1A). Similar to results in a previous report [6], inflammasome activators induced various levels of ROS inside cells, especially ATP (Fig. 1B and C). Given that GSH acts as a major antioxidant in mammalian cells by balancing the cellular redox status, we performed a parallel study with liquid chromatography-tandem mass spectrometry (LC-MS/MS) to quantify changes in intracellular GSH levels. We found that GSH levels in BMDMs dropped significantly after ATP treatment (Fig. 1D). We then confirmed our findings by conducting the same experiments with cells of the murine macrophage cell line J774.1. We observed IL-1β production and ROS generation tendencies in NLRP3 inflammasome-activated J774.1 cells that were similar to those in BMDMs (Fig. 1E–G). As an important result, the intracellular GSH level was nearly entirely depleted after ATP stimulation in LPS-primed J774.1 cells (Fig. 1H and S1). On the other hand, no such remarkable GSH depletion was observed for J7744.1 cells treated with nigericin, *S*. Typhimurium LT2, and poly(dA:dT) (Fig. 1H and S2).

We next investigated whether GSH depletion was sufficient for NLRP3 inflammasome activation. We treated J774.1 cells with l-buthionine-(*S*,*R*)-sulfoximine (BSO) (which is a γ-glutamylcysteine synthetase inhibitor) for 16 h (Fig. S3A). Under these conditions, GSH depletion occurred to an extent that was similar to that induced by ATP exposure (Fig. S3A). As Fig. S3B indicates, BSO treatment of LPS-primed cells failed to induce IL-1β production. This result suggests that GSH depletion alone was insufficient to activate NLRP3 inflammasome.

**Fig. 2:**
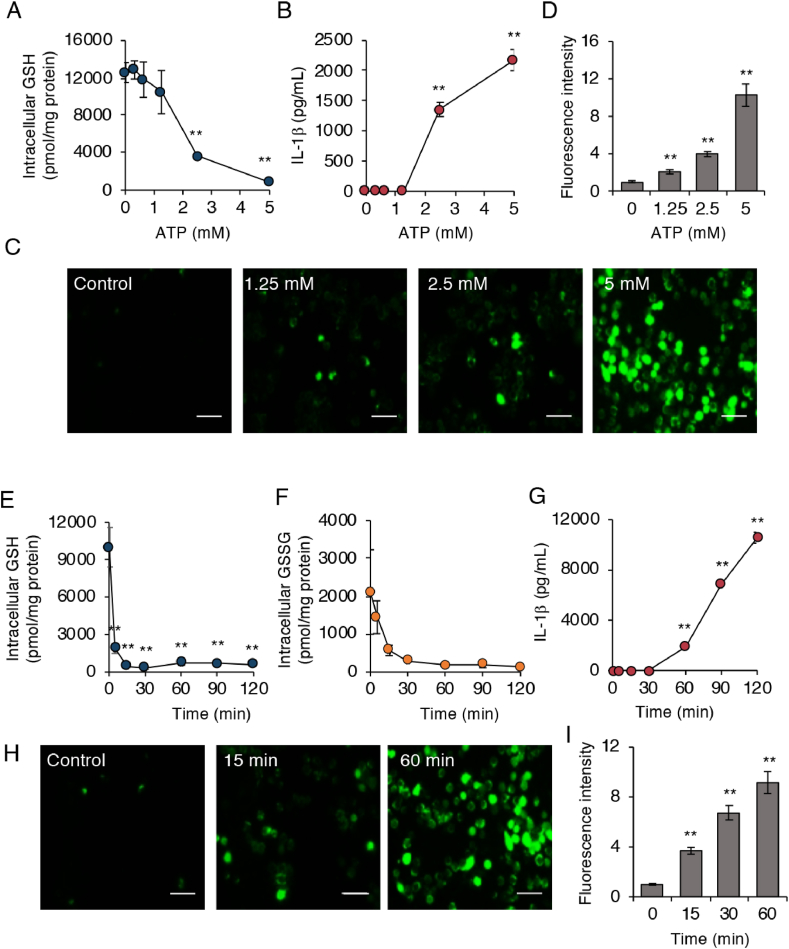
**GSH depletion after ATP treatment occurs in advance of NLRP3 inflammasome activation.** (A–D) J774.1 cells were primed with LPS (100 ng/mL) for 5 h, followed by stimulation with ATP at the indicated concentrations. (A) Intracellular GSH levels were quantified by means of LC-MS/MS. (B) IL-1β levels in culture supernatants and (C) ROS generation was determined by using ELISA and DCF-DA, respectively. (D) Quantitative data for results in (C). (E–I) LPS-primed J774.1 cells were treated with ATP (5 mM) for 5, 15, 30, 60, 90, or 120 min. Results for intracellular GSH (E), GSSG (F), IL-1β production (G), and (H) ROS generation in J774.1 cells are shown. (I) Quantitative data for results in (H). Scale bars: 50 μm. Data represent means ± SD (n = 3). **p < 0.01.

We then analyzed the effects of ATP exposure on GSH depletion, particularly in relation to NLRP3 inflammasome activation and ROS generation. Fig. 2A shows significantly lower GSH levels at ATP concentrations higher than 1.25 mM compared with those at lower ATP concentrations. We detected IL-1β production when LPS-primed cells were challenged with ATP at concentrations higher than 1.25 mM (Fig. 2B). We also found that ROS production in J774.1 cells depended on the ATP dose (Fig. 2C and D). These data suggest that extent of the GSH decrease correlated well with the reductions in ROS production and IL-1β production.

A time course study showed that the GSH decrease occurred quite rapidly just 5 min after ATP exposure at a fixed concentration of 5 mM ATP (Fig. 2E). Our metabolomic analyses also demonstrated an identical time-dependent decrease for GSH-related molecules including GSSG (Fig. 2F), glutathione persulfide (GSSH), and glutathione polysulfide (GSSSH) (Fig. S4). Cysteine (CysSH), a source for GSH biosynthesis, decreased after GSH depletion (Fig. S4A). However, the GSH biosynthesis intermediate γ-glutamylcysteine (γ-Glu-Cys) increased after ATP exposure (Fig. S4G). IL-1β production, however, became clear 60 min after ATP exposure (Fig. 2G). As seen in Fig. 2H and I, significant ROS generation occurred beginning 15 min of ATP stimulation. These data suggest that the GSH decrease occurred before ROS production and IL-1β production.

Previous studies described the effects of NOX-derived ROS on NLRP3 inflammasome activation [7,25]. In agreement with those reports, we found that IL-1β production by cells stimulated with LPS/ATP was suppressed by treatment with apocynin, a NOX inhibitor (Fig. S3C). ROS production was somewhat inhibited by apocynin (Fig. S3D), which suggests the involvement of an ROS source other than NOX, such as mitochondria. In sharp contrast, the reduced GSH levels caused by ATP exposure did not recover after apocynin treatment (Fig. S3E). These data suggest that GSH depletion and ROS generation were induced separately by ATP exposure.

### 3.2 Reduced GSH levels are mediated by the P2X7 receptor

**Fig. 3:**
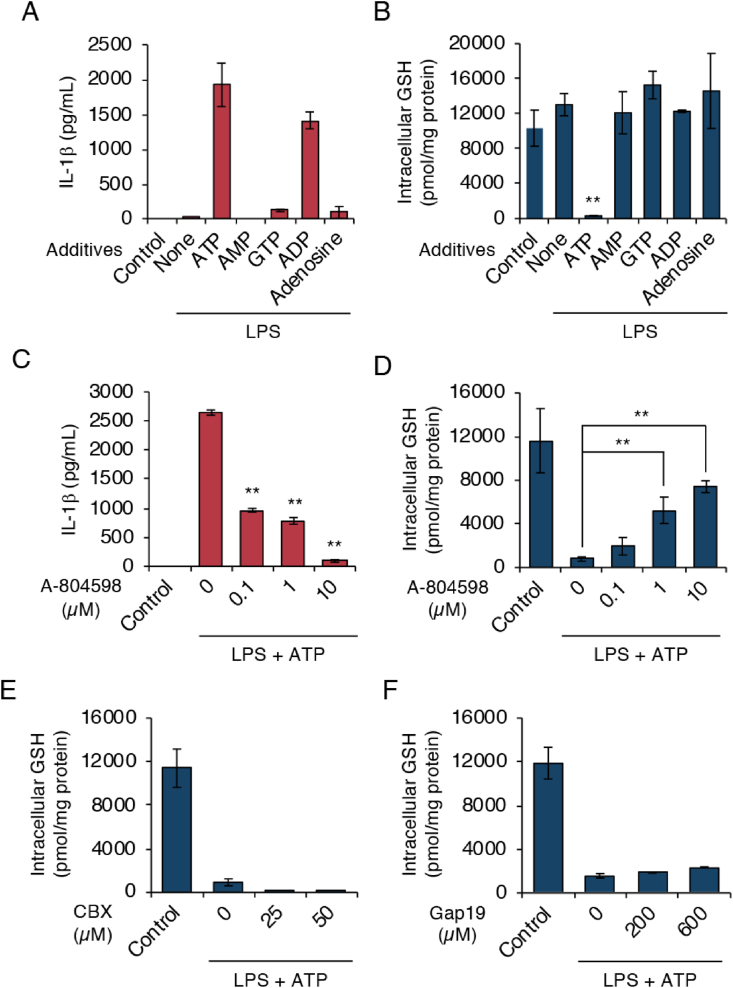
**GSH depletion depends on P2X7 receptor signaling.** (A and B) J774.1 cells were primed with LPS (100 ng/mL) for 5 h and then stimulated with the indicated nucleotides or nucleoside (5 mM) for 1 h. (C–F) J774.1 cells were pre-incubated with A-804598, CBX, or Gap19 at the indicated concentrations before being primed for 1 h with LPS (100 ng/mL), after which they were stimulated for 1 h with ATP (5 mM). (A and C) Levels of IL-1β in culture supernatants were measured by means of ELISA. (B and D-F) LC-MS/MS was used for to determine intracellular GSH levels. Controls were cells not treated with either LPS or stimulus. Data represent means ± SD (n = 3). **p < 0.01.

Various nucleotides reportedly triggered NLRP3 inflammasome activation [26]. To examine the possibility that hydrolyzed products of ATP induced the GSH depletion, we treated LPS-primed J774.1 cells with ATP or its related molecules. Both ATP and ADP induced measurable amounts of IL-1β (Fig. 3A). We also found that both GTP and adenosine activated the NLRP3 inflammasome, but only slightly (Fig. 3A). However, a reduction in GSH levels occurred only when LPS-primed J774.1 cells were exposed to ATP (Fig. 3B).

ATP binding to the P2X7 receptor opens two types of permeable pores—pannexin channels and connexin channels—which are responsible for downstream signaling events [27]. We therefore analyzed whether the P2X7 receptor mediated ATP-induced GSH depletion by using A-804598 (a P2X7 receptor antagonist). Fig. 3C demonstrates that A-804598 dose-dependently inhibited NLRP3 inflammasome activation induced by ATP exposure. ATP-induced GSH depletion stopped when cells were treated with ATP in the presence of A-804598 (Fig. 3D). These results clearly suggest that the P2X7 receptor mediated ATP-induced GSH depletion during NLRP3 inflammasome activation. We also studied the involvement of the pannexin-1 channel and the connexin channel on ATP-induced GSH depletion by using their inhibitors carbenoxolone (CBX) (pannexin-1 channel blocker) and Gap19 (connexin 43 channel blocker), respectively. Neither CBX nor Gap19 treatment affected ATP-induced GSH depletion (Fig. 3E and F), which suggests that GSH depletion occurred independently of these channels.

### 3.3 ATP exposure triggers intracellular GSH efflux

**Fig. 4:**
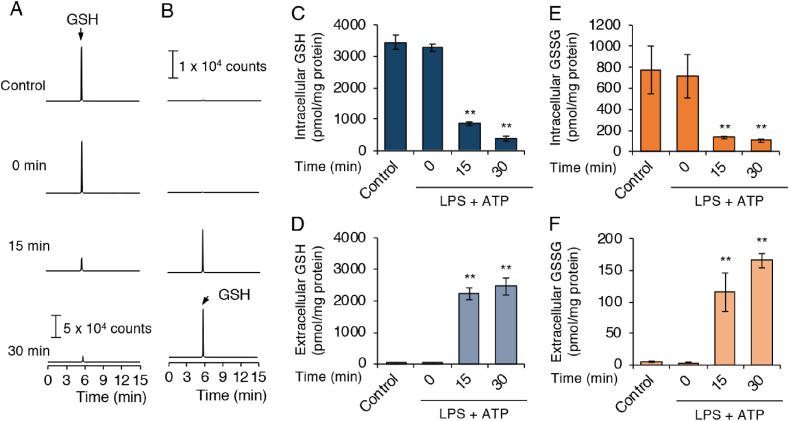
**ATP treatment induces GSH efflux.** LPS (100 ng/mL) was added to J774.1 cell cultures and after 5 h of incubation, cells were stimulated with ATP (5 mM) for the indicated time periods. Representative extracted ion chromatograms (EIC) for (A) intracellular GSH and (B) extracellular GSH after NLRP3 inflammasome activation in J774.1 cells. Intracellular levels of GSH (C), GSSG (E), and extracellular levels of GSH (D), GSSG (F) were quantified via LC-MS/MS. Controls were cells not treated with either LPS or ATP. Data represent means ± SD (n = 3). **p < 0.01.

To determine whether ATP exposure could stimulate GSH efflux from cytosol to the extracellular space, we analyzed GSH levels in culture supernatants. An MS-based analysis showed the clear presence of GSH in supernatants of cultured J774.1 cells after ATP treatments of both 15 and 30 min (Fig. 4). The GSH levels in the culture supernatants were almost equivalent to the decreased intracellular GSH levels in J774.1 cells after ATP exposure (Fig. 4A–D). We also found the decrease of intracellular GSSG and the increase of GSSG in the culture supernatants after ATP treatments (Fig. 4E and F). These data strongly suggest that ATP exposure stimulated the efflux of GSH and GSSG, implicating in the depletion of these molecules.

**Fig. 5:**
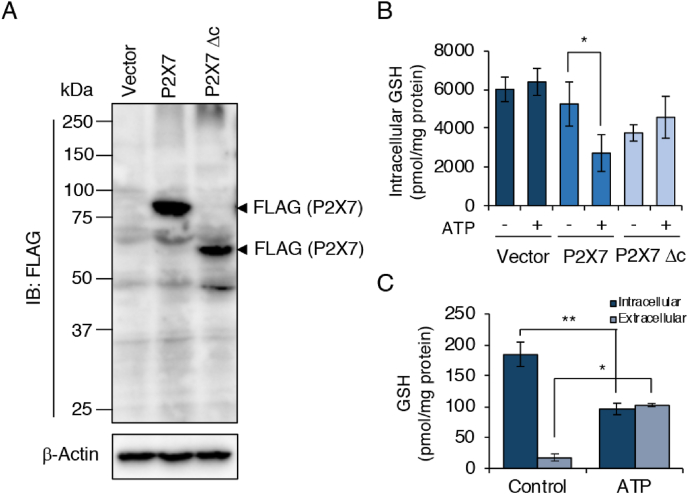
**ATP treatment induces GSH efflux in P2X7 receptor-expressing HEK293FT cells.** HEK293FT cells were transfected with empty vector, flag-pcDNA3-P2X7, or flag-pcDNA3-Δc-P2X7 for 48 h. Transfected HEK293FT cells were then treated with ATP (5 mM) for 1 h. (A) Western blotting showing flag expression in HEK293FT cells after transfection. Lysates were prepared from HEK293FT cells after transfection for 48 h. (B) Intracellular GSH levels in transfected HEK293FT cells were determined by using LC-MS/MS after 1 h of ATP stimulation. (C) LC-MS/MS measurement of intracellular and extracellular GSH in P2X7-expressing HEK293FT cells after ATP stimulation. Controls were cells not treated with ATP. Data represent means ± SD (n = 3). *p < 0.05; **p < 0.01.

To verify the role of the ATP-P2X7 receptor axis in GSH efflux, we established P2X7-expressing HEK293FT cells by transfecting either flag-P2X7-expressing (wild type) or Δc-P2X7-expressing (mutant) vectors (Fig. 5A). We observed that GSH levels decreased after ATP treatment only in P2X7-expressing HEK293FT cells, not in Δc-P2X7-expressing HEK293FT cells or in control cells (Fig. 5B). In addition, we successfully detected GSH in culture supernatants obtained from P2X7-expressing HEK293FT cells after ATP treatment (Fig. 5C).

### 3.4 Exogenously added GSH ameliorates GSH depletion and suppresses IL-1β release

**Fig. 6:**
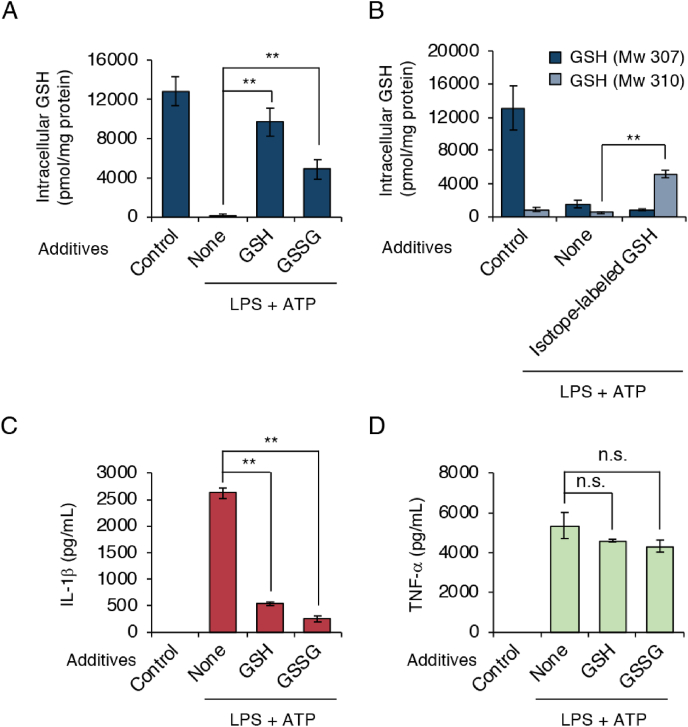
**Exogenous addition of GSH inhibits GSH depletion and IL-1β production.** J774.1 cells were primed with LPS (100 ng/mL) for 5 h, after which they were stimulated for 1 h with ATP (5 mM) in the absence or presence of additives (10 mM) as indicated. (A) Intracellular GSH levels were quantified by means of LC-MS/MS. (B) Effect of the addition of isotope-labeled GSH on intracellular GSH levels in J774.1 cells. Isotope-labeled GSH (Mw 310, 5 mM) was added to culture medium during ATP stimulation. Intracellular levels of GSH having Mw of 307 and 310 were determined. (C) IL-1β levels in culture supernatants were measured by using ELISA. (D) J774.1 cells were primed with LPS (100 ng/mL) for 5 h, followed by stimulation with ATP (5 mM) in the absence or presence of GSH (5 mM) and GSSG (5 mM). TNF-α levels in culture supernatants were quantified 1 h later by using ELISA. Controls were cells not treated with either LPS or stimulus. Data represent means ± SD (n = 3). *p < 0.05; **p < 0.01; n.s., not significant.

We studied whether exogenous additions of GSH and GSSG affected intracellular GSH levels as well as NLRP3 inflammasome activation. We added GSH or GSSG to cells when LPS-primed cells were treated with ATP. As shown in Fig. 6A, exogenous additions of GSH and GSSG increased intracellular GSH levels. When isotope-labeled GSH (*glycine*-^13^C_2_, ^15^N; Mw 310; 3 mass unit larger than endogenous GSH) was added exogenously, increase of GSH having Mw of 310 was clearly detected in cells (Fig. 6B). This data suggests that GSH added extracellularly was incorporated into cells that compensated for the GSH depletion induced by ATP exposure. We observed that exogenous additions of GSH and GSSG suppressed the IL-1β production as well (Fig. 6C). In contrast, TNF-α production was not affected by exogenous additions of GSH and GSSG (Fig. 6D). These data suggest that GSH depletion acts as an upstream signal in NLRP3 inflammasome activation.

We next evaluated the previous result implicating GSH transporters as possible candidates inducing GSH efflux. We added MK571, an inhibitor of multidrug resistance proteins (common GSH transporters), to cells during activation of the NLRP3 inflammasome. However, we observed no marked difference in GSH efflux after ATP treatment between cells either treated with MK571 or not treated, as Fig. S5A demonstrates. We also found that both *p*-aminohippuric acid (PAH) [inhibitor of organic anion transporters (OATs)] and rifamycin SV (inhibitor of OAT polypeptides) failed to prevent ATP-induced GSH efflux (Figs. S5B and S5C). Similar results were observed when cells were incubated with inhibitors of other ATP-binding cassette (ABC) transporters, including glibenclamide (cystic fibrosis transmembrane conductance regulator inhibitor) and Ko143 (ABCG2 inhibitor) (Figs. S5D and S5E). Moreover, two possible channel blockers—phloretin (volume-regulated anion channel blocker) and 4,4ʹ-diisothiocyanatostilbene-2,2ʹ-disulfonic acid disodium salt hydrate (DIDS) (maxi-channel blocker)—did not affect GSH efflux in NLRP3 inflammasome-activated J774.1 cells (Figs. S5F and S5G). ATP-induced GSH efflux was not associated with the endoplasmic reticulum (ER)-Golgi transport system, as supported by the finding of no substantial alteration of GSH efflux when cells were treated with ATP in the presence of brefeldin A (BFA) (ER-Golgi protein trafficking inhibitor) (Fig. S5H).

Upon activation of NLRP3 inflammasome, cytosolic GSDMD is proteolytically cleaved by caspase-1 to form GSDMD N-terminal fragment. The N-terminal fragment is then bound to plasma membrane to form GSDMD pore that facilitate the release of intracellular components including ATP and IL-1β [28]. We examined the implication of GSDMD pore formation on ATP-induced GSH efflux. Expression of GSDMD was reduced by means of siRNA knockdown (Fig. S6A). ATP-induced GSH efflux was determined in GSDMD knockdown cells in a similar extent to control siRNA treated cell, suggesting that GSDMD pore formation was not responsible for ATP-induced GSH efflux (Fig. S6B). Although we did not identify the GSH exporter involved in ATP-induced GSH efflux, these data indicate that an unconventional GSH transport mechanism may exist upstream of NLRP3 inflammasome activation.

### 3.5 GSH efflux is a distinct independent event associated with potassium efflux during NLRP3 inflammasome activation

**Fig. 7:**
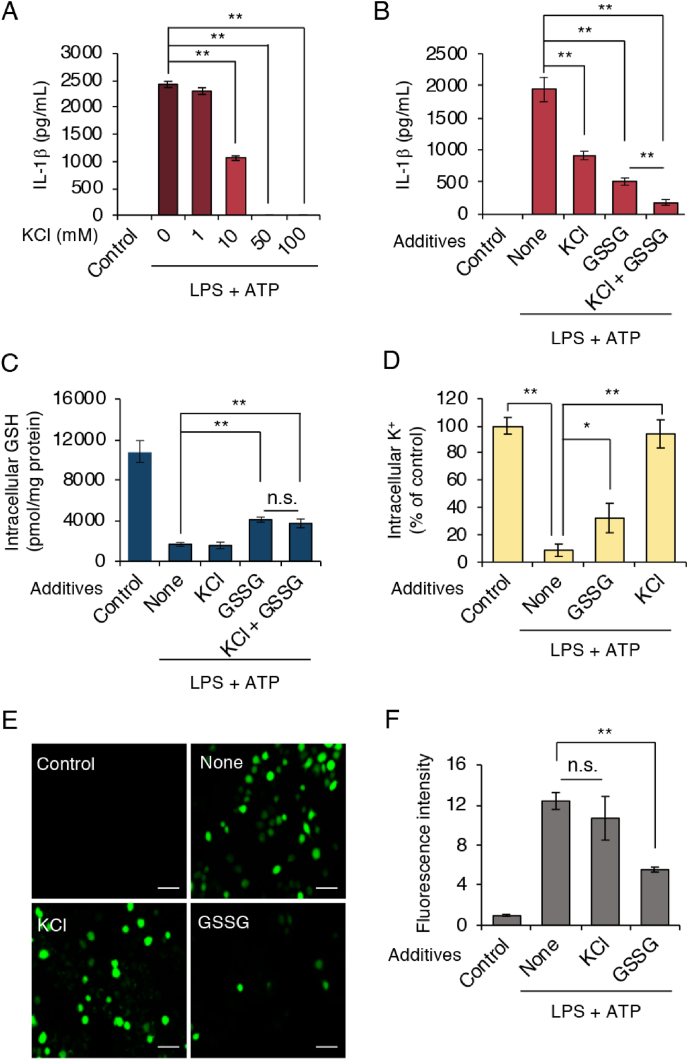
**Exogenous addition of GSH and GSSG inactivate the NLRP3 inflammasome independently of K**^**+**^. (A) J774.1 cells were primed with LPS (100 ng/mL) for 5 h, followed by ATP (5 mM) stimulation in the absence or presence of KCl at the indicated concentrations for 1 h. IL-1β production was determined by using ELISA. (B–F) LPS-primed J774.1 cells were stimulated with ATP (5 mM) in the absence or presence of GSSG (5 mM) and KCl (10 mM) for 1 h. (B) IL-1β and (C) intracellular GSH were quantified by using ELISA and LC-MS/MS, respectively. (D) Concentrations of intracellular K^+^ were measured via a FluxOR II Green Potassium Ion Channel Assay Kit. (E) ROS generation was determined by means of a DCF-DA probe. (F) Quantitative data for results in (E). Scale bars: 50 μm. Data represent means ± SD (n = 3). *p < 0.05; **p < 0.01; n.s., not significant. (For interpretation of the references to colour in this figure legend, the reader is referred to the Web version of this article.)

K^+^ efflux is a well-known common event during NLRP3 inflammasome activation, and inhibition of K^+^ efflux blocked NLRP3 inflammasome activation [29]. Therefore, our next analysis involved study of the association between K^+^ efflux and GSH efflux. We activated the NLRP3 inflammasome in J774.1 cells by using ATP in either the absence or presence of different concentrations of potassium chloride (KCl). Our results corroborated previous findings: we observed a dose-dependent inhibition of IL-1β production induced by exogenous addition of KCl, with complete inhibition of IL-1β production at 50 mM extracellular K^+^ (Fig. 7A). Because addition of 10 mM K^+^ inhibited IL-1β production by approximately 50%, we studied the effects of the addition of both GSSG and 10 mM KCl. Fig. 7B shows that IL-1β production decreased by 50% when the NLRP3 inflammasome was activated in the presence of 10 mM KCl. GSSG treatment demonstrated a stronger suppression of IL-1β production, with this suppression being strengthened when cells were treated with KCl together with GSSG. Under the same experimental conditions, LC-MS/MS-based analysis again confirmed our previous findings that exogenous GSSG treatment significantly ameliorated ATP-induced GSH efflux (Fig. 7C). GSH efflux, however, was unaffected by KCl or the KCl and GSSG combination (Fig. 7C). We also demonstrated that 10 mM extracellular KCl almost entirely inhibited K^+^ efflux during NLRP3 inflammasome activation, whereas exogenous addition of GSSG only partially halted this K^+^ efflux (Fig. 7D). In contrast, exogenous KCl had almost no effect on ROS generation under equivalent conditions (Fig. 7E and F). However, ROS generation was strongly suppressed by adding GSSG exogenously (Fig. 7E and F). From these data we conclude that GSH efflux was a K^+^ efflux-independent event during NLRP3 inflammasome activation.

### 3.6 Intracellular GSH level affects NLRP3 inflammasome complex assembly

**Fig. 8:**
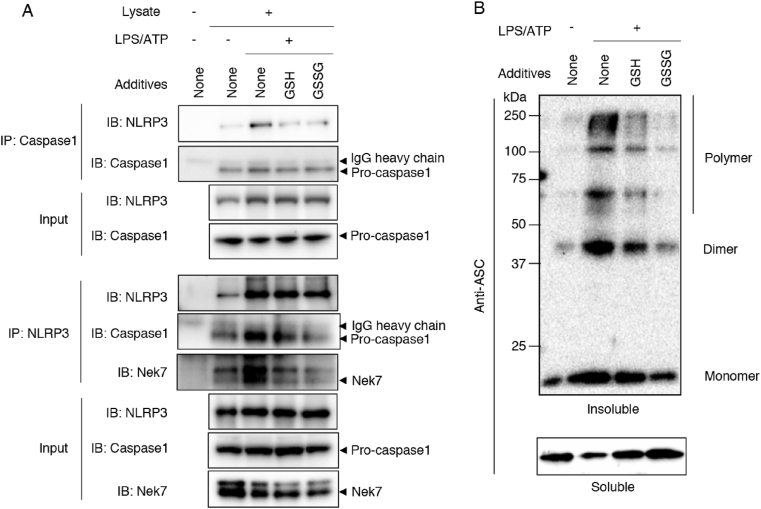
**Exogenous addition of GSH and GSSG suppresses NLRP3 inflammasome complex assembly.** (A) J774.1 cells were primed with LPS (100 ng/mL) for 5 h, followed by stimulation with ATP (5 mM) in the absence or presence of GSH (5 mM) and GSSG (5 mM) for 1.5 h. Samples were then harvested and immunoprecipitated by using the indicated antibodies. Immunoprecipitated complexes were analyzed via Western blotting, with results shown here. (B) LPS-primed BMDMs were stimulated under the same conditions as those used in (A). Cells were lysed in lysis buffer containing NP-40. The NP-40-insoluble fraction was crosslinked by using BS3. Results of Western blotting of ASC expression in both NP-40-soluble and NP-40-insoluble fractions are shown. Data represent means ± SD (n = 3). IP, immunoprecipitation; IB, immunoblotting.

To investigate the regulatory mechanisms involved in GSH efflux-mediated NLRP3 inflammasome activation, we performed co-immunoprecipitation and ASC oligomerization assays to analyze the NLRP3 inflammasome complex assembly. Fig. 8A illustrates the successful pull-down of the NLRP3 inflammasome complex from stimulated cells via anti-caspase-1 and anti-NLRP3 antibodies. Note that exogenously added GSH and GSSG interfered with NLRP3 and caspase-1 binding. Nek7 binding to NLRP3 was also significantly inhibited by exogenous addition of GSH or GSSG (Fig. 8A).

As seen in Fig. 8B, ASC oligomerization was induced by treatment with LPS plus ATP. ASC oligomerization was strongly inhibited when cells were treated with exogenous GSH as well as GSSG. Collectively, these data suggest that intracellular GSH had negative impacts on protein assembly of the NLRP3 inflammasome during the activation process.

### 3.7 GSH efflux as a potential target for NLRP3 inflammasome-associated diseases

**Fig. 9:**
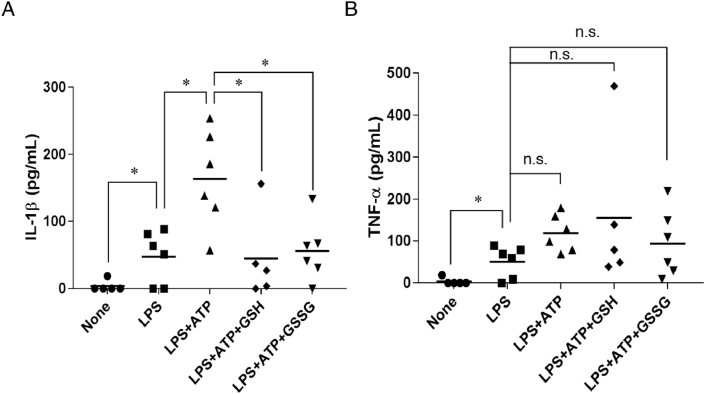
**Administration of GSH and GSSG suppresses IL-1β production in mice.** Mice received either LPS (2 μg/kg body weight) or saline intraperitoneally for 1.5 h. The mice were subsequently injected intraperitoneally with ATP (50 μmol/kg body weight) in either the absence or the presence of GSH (50 μmol/kg body weight) or GSSG (50 μmol/kg body weight). Mice injected with saline only served as a negative control. One hour after the ATP injection, levels of (A) IL-1β and (B) TNF-α in serum were measured by using ELISA. Data represent means ± SD (n = 6). *p < 0.05; n.s., not significant.

In view of the pathogenic role of IL-1β hyperproduction in numerous NLRP3 inflammasome-associated diseases, we investigated the therapeutic potential of GSH in a mouse model of cytokine production induced by LPS and ATP [20]. In the present experimental setting, we detected no appreciable IL-1β in serum samples obtained from mice that received saline. However, LPS administration led to a considerable increase in IL-1β levels in serum. In comparison, an even higher level of IL-1β occurred in the serum of mice receiving intraperitoneal ATP after LPS administration. As expected, both GSH treatment and GSSG treatment significantly suppressed IL-1β production in mice receiving LPS and ATP, to the levels comparable to those in mice receiving LPS alone (Fig. 9A). We also studied TNF-α production in this animal model. Fig. 9B illustrates that similar levels of TNF-α were induced when mice received LPS intraperitoneally either alone or as a combination of LPS and ATP. Neither GSH treatment nor GSSG treatment ameliorated TNF-α production in mice (Fig. 9B). These findings thus suggest that GSH may exert a prophylactic effect against inflammatory diseases caused by excessive and dysregulated activation of the NLRP3 inflammasome induced by ATP exposure.

## 4 Discussion

Although a critical role of ROS generation in the activation of the NLRP3 inflammasome was proposed more than a decade ago, consensus on the precise mechanism of this effect is still lacking [30]. Given that multiple sources of ROS exist inside cells, we, coming from the opposite perspective, evaluated the redox status on NLRP3 inflammasome activation by focusing on the antioxidant molecule GSH. We found that intracellular GSH almost entirely disappeared when macrophages received ATP treatment alone (Fig. 1 and S1), even if nigericin-induced or poly(dA:dT)-induced ROS were similar to ATP-induced ROS in BMDMs (Fig. 1). Cruz et al. previously reported that treatment of macrophages with ATP resulted in ROS-dependent activation of the NLRP3 inflammasome [31]. After confirmation of this finding via our experimental model, we proceeded to demonstrate that neither a low dose of ROS nor a short time exposure to ROS was sufficient to induce NLRP3 inflammasome-mediated IL-1β production. As a more important result, our data showed that GSH depletion occurred rapidly in response to a high ATP dose (Fig. 2). These results suggested that ATP-dependent NLRP3 inflammasome activation may involve a cooperative loop between GSH depletion and ROS generation. In our experiment with BSO-treated J774.1 cells, GSH depletion via inhibition of GSH biosynthesis instead limited NLRP3 inflammasome-mediated IL-1β production (Figs. S3A and S3B). This result reinforces the importance of ATP-induced GSH depletion in ROS-dependent NLRP3 inflammasome activation.

Nucleotides have been demonstrated to promote high ROS production and IL-1β secretion via various purinergic receptors [31]. Indeed, we detected IL-1β production in the culture supernatant of LPS-primed J774.1 cells treated with several nucleotides including ATP, GTP, and ADP, and adenosine (Fig. 3A). Nevertheless, LC-MS/MS analysis showed that GSH depletion responded only to ATP stimulation (Fig. 3B). In addition, we found that A-804598, a P2X7 receptor antagonist, dose-dependently rescued GSH depletion (Fig. 3C and D). Our data thus definitively demonstrated that the ATP-induced GSH depletion was mediated by the P2X7 receptor. The key role of this P2X7 receptor in this phenomenon was confirmed by the finding that ATP treatment reduced the intracellular GSH level in P2X7 receptor-expressing HEK293FT cells (Fig. 5A and B). When we used l-methionine- and l-cystine-free medium, we observed a similar increase in GSH levels in the extracellular space after ATP treatment, which suggests that ATP stimulation led to GSH efflux (Fig. 4, Fig. 5C); that is, GSH function as an ROS scavenger was limited during activation of the NLRP3 inflammasome in response to ATP exposure. To our best knowledge, these data are the first to show the occurrence of GSH efflux as a proximal upstream event for NLRP3 inflammasome activation. Thus, an intriguing possibility is that a rapid GSH efflux may promote an imbalance in intracellular redox status and an enhancement of ROS accumulation, both of which may be a leading mechanism for activation of the NLRP3 inflammasome via cellular ROS.

Recent study by Wang et al. demonstrated that ATP-induced activation of P2X7 receptor and NLRP3 inflammasome was promoted by Paxillin [32]. They found that extracellular ATP induced phosphorylation of Paxillin that led to the formation of P2X7 receptor-Paxillin-NLRP3 complex. The complex formation then facilitated NLRP3 inflammasome complex assembly through deubiquitination of NLRP3 [32]. Effects of ATP-induced GSH efflux on Paxillin phosphorylation are currently unknown, and warrant further elucidation to understand the molecular mechanisms how GSH efflux activates NLRP3 inflammasome.

ATP exposure induced NOX complex assembly and increased complex activity, which was critical for ROS generation and IL-1β secretion in monocyte THP-1 cells [25]. Also, NOX inhibitor treatment inhibited ATP-induced caspase-1 and IL-1β processing in these same cells [25]. Our data corroborated those findings by demonstrating that inhibition of NOX activity via a pharmacological inhibitor (apocynin) significantly suppressed IL-1β production in macrophages (Fig. S3C). We also noted that apocynin treatment had no effect on ATP-induced GSH efflux (Fig. S3E). This result may be attributed to the fact that GSH efflux induced by ATP probably occurred before NOX activation and ROS accumulation. Alternatively, mitochondria are a well-known major source of cellular ROS. Numerous studies demonstrated that NLRP3 inflammasome activation relied on mROS [13]. Thompson et al. reported that asbestos promoted NLRP3 inflammasome activation via mROS generation [33]; they also noted that asbestos exposure coincided with GSH depletion and γ-Glu-Cys synthetase upregulation [34]. One study reported a causal link between the exposure of human lung epithelial cells to asbestos and GSH efflux, although this finding was not well documented [35]. Similarly, we observed that ATP stimulation induced a rapid GSH efflux, with an increase in intracellular γ-Glu-Cys levels (Fig. 2, Fig. 4, and S4G). This finding suggests the existence of a γ-Glu-Cys synthetase-dependent compensatory mechanism that would restore the reduced GSH levels. The internalization of asbestos by macrophages resulted in NLRP3 inflammasome activation and cell death, similar to the effects of other crystals or nanoparticles [7]. Given that ATP release represents a physiological condition that occurs only when cells are dying or platelets are degranulating [36], we can logically conclude that GSH efflux may be a common event used by ROS derived from different sources to activate the NLRP3 inflammasome.

Most studies have linked GSH depletion to the progression of cell death and have provided several candidates for associated GSH transporters [37]. In an attempt to identify the mechanisms involved in GSH efflux, we examined the effects of inhibitors that are known to block ATP-induced GSH efflux (Figs. S5A–S5E). We also performed experiments to test different channels that may participate in ATP-induced GSH efflux via channel blockers (Fig. 3E and F, and S5F–S5H). Unfortunately, we could not identify the specific GSH transporter involved in ATP-induced GSH efflux; however, we deduced that GSH efflux induced by ATP was a distinct, unconventional mechanism. One possibility is the P2X7 receptor itself, since previous reports have indicated that overstimulation of P2X7 receptors may lead to pore formation for large molecules [38]. Consistent with this, we observed that ATP-induced GSH efflux was indeed determined in P2X7 receptor-expressing HEK293 FT cells (Fig. 5). Also, it is known that NLRP3 inflammasome activation leads to GSDMD pore formation, that allows release of intracellular components including ATP and IL-1β [28]. Our data, however, suggested that GSDMD pore may not play a role in ATP-induced GSH efflux (Fig. S6). GSH depletion has also been implicated in other processes and disorders, such as apoptosis, cytokine production, T cell activation, HIV infection, and inflammation [39,40]. We also examined the effects of ATP treatment on cell viability (Fig. S7). LPS and ATP treatment decreased cell viability compared to untreated control (Fig. S7). Exogenous additions of GSH and GSSG partially rescued ATP-induced cell death. In view of these data, additional studies are warranted to determine the signaling cascade as well as GSH transporters/pores involved in the ATP-dependent GSH efflux and the exact role of released GSH in overall physiology [41].

Our results for isotope-labeled GSH suggest that macrophages can internalize exogenous GSH during ATP-dependent GSH efflux. We also found that the exogenous addition of either GSH or GSSG significantly inhibited production of IL-1β, but not TNF-α, with a concomitant increase in intracellular GSH levels (Fig. 6A, C, and 6D). To better understand these mechanisms, we analyzed the effect of GSH on formation of the NLRP3 inflammasome complex. We found that exogenous GSH or GSSG treatment strongly inhibited the interaction between NLRP3 and Nek7, and between NLRP3 and caspase-1 (Fig. 8A). ASC assembly was also significantly attenuated by both GSH and GSSG added exogenously (Fig. 8B). Therefore, our findings demonstrate that intracellular GSH functions as a negative regulator of the NLRP3 inflammasome.

K^+^ efflux is widely accepted as a common upstream signal for NLRP3 inflammasome activation, and high extracellular concentrations of K^+^ can block NLRP3 inflammasome activation [29]. In agreement with this information, we found that ATP-mediated NLRP3 inflammasome activation as assessed by IL-1β production was inhibited in cells that were incubated with high KCl concentrations in the culture medium (Fig. 7A). We found enhanced inhibition of IL-1β production by KCl when cells received a combination of KCl and GSSG, whereas we did not observe an effect of KCl on intracellular GSH levels or ROS accumulation (Fig. 7B, C, 7E, and 7F). Treating cells with KCl almost fully restored the original intracellular K^+^ levels. However, exogenous addition of GSSG seemed to increase the concentration of intracellular K^+^ only slightly (Fig. 7D). Collectively, these results suggest an independence between K^+^ efflux and the inhibitory effect of GSH on the NLRP3 inflammasome.

Although both ATP and nigericin induce K^+^ efflux as an essential signal for NLRP3 inflammasome activation, these two substances induce K^+^ efflux by mechanistically distinct pathways [42]. Binding of ATP to P2X7 receptor results in opening of cation channels to induce K^+^ efflux [42]. As discussed above, GSH may be extruded through such P2X7 receptor-mediated channel opening. In contrast to ATP/P2X7 receptor system, nigericin can act as a K^+^/H^+^ antiport ionophore that exchanges K^+^ for H^+^ across cell membranes [42]. Our data suggest thus that GSH efflux occurs in a stimulant dependent manner, and nigericin could activate NLRP3 inflammasome without induction of GSH efflux (Fig. 1D and H).

The natural next question is how precisely does GSH regulate the NLRP3 inflammasome. Thioredoxin (TRX) is ubiquitously expressed in mammalian cells and, together with thioredoxin reductase and NADPH, is responsible for maintenance of the redox milieu [43]. Under physiological conditions, thioredoxin-interacting protein (TXNIP) is believed to strictly control TRX activity via a redox-dependent interaction [44]. Several studies reported that dissociation of TXNIP from TRX occurred in response to oxidative conditions induced by phagocytosis of particles, which allowed free TXNIP to bind with NLRP3 and activate the NLRP3 inflammasome [45]. This finding suggests that intracellular GSH exerted its antioxidant activity to inhibit dissociation of TXNIP and TRX by modulating the cellular redox balance. ROS can promote the oxidative S-glutathionylation of proteins via reactions with the cysteine amino acids of proteins [46]. Meissner et al. found that superoxide dismutase 1 regulated caspase-1 activation via glutathionylation of cysteine residues of caspase-1 [18]. The anti-inflammatory effect of curcumin was associated with S-glutathionylation of the key component of the NLRP3 inflammasome [47]. More recently, glutathionylation of Nek7, another important NLRP3 inflammasome component, was found to limit NLRP3 inflammasome activation [17]. In that study, Hughes et al. showed that glutathione transferase omega1-1 deglutathionylated NEK7, that promoted NLRP3 inflammasome activation [17]. As we mentioned above, GSH efflux-enhanced ROS accumulation can create a suitable cellular environment for protein S-glutathionylation. These findings may provide useful information for the identification of target proteins or signaling cascades in our experimental system. Continued study is warranted to determine the detailed molecular mechanisms involved in GSH-regulated inactivation of the NLRP3 inflammasome by identifying which target molecules are regulated by GSH.

*In vivo* experiments demonstrated that treatment with both GSH and GSSG protected mice from dysregulation of the NLRP3 inflammasome, as evidenced by a significant reduction in IL-1β production but not TNF-α production (Fig. 9). In fact, clinical strategies to restore GSH levels are not without precedent [[48], [49], [50]]. However, our data suggest that GSH and/or GSSG, as potential therapeutic agents, may be used to treat NLRP3 inflammasome-related inflammatory diseases.

In summary, we provide evidence, for the first time, that GSH efflux occurs in the proximal upstream region of the NLRP3 inflammasome in response to ATP exposure. This ATP-induced GSH efflux leads to an accumulation of cellular ROS and an imbalance in redox status with subsequent activation of the NLRP3 inflammasome. The P2X7 receptor antagonist markedly inhibited both NLRP3 inflammasome-mediated IL-1β production and GSH efflux, which suggests that the P2X7 receptor mediates ATP-dependent GSH efflux. As an important result, exogenous GSH and GSSG strongly inhibited assembly of the NLRP3 inflammasome complex and secretion of IL-1β by increasing intracellular levels of GSH. An *in vivo* study also demonstrated that production of IL-1β but not of TNF-α was significantly ameliorated by intraperitoneal injection of GSH or GSSG. Our findings may resolve the controversy regarding the role of ROS in the NLRP3 inflammasome by providing insight into redox-dependent regulation of NLRP3 inflammasome activation and hence they may lead to potential therapeutic targets in NLRP3 inflammasome-associated disorders.

## Author contributions

T.S. and T.Z. designed the study. T.Z., H.T., W.I., K.O., and K.T. conducted the experiments. T.Z., H.T., and K.O. analyzed the data. T.S. and T.Z. wrote the manuscript. T.S. and T.A. edited the manuscript.

## Declaration of competing interest

The authors declare no competing interests.
