# Supplementary material for: ATP exposure stimulates glutathione efflux as a necessary switch for NLRP3 inflammasome activation
Source: Redox Biol. 2021 Mar 10;41:101930. doi: 10.1016/j.redox.2021.101930 (PMC7995658; doi:10.1016/j.redox.2021.101930)
Supplement: Multimedia component 1 [file mmc1.pdf]

Supplementary Information:

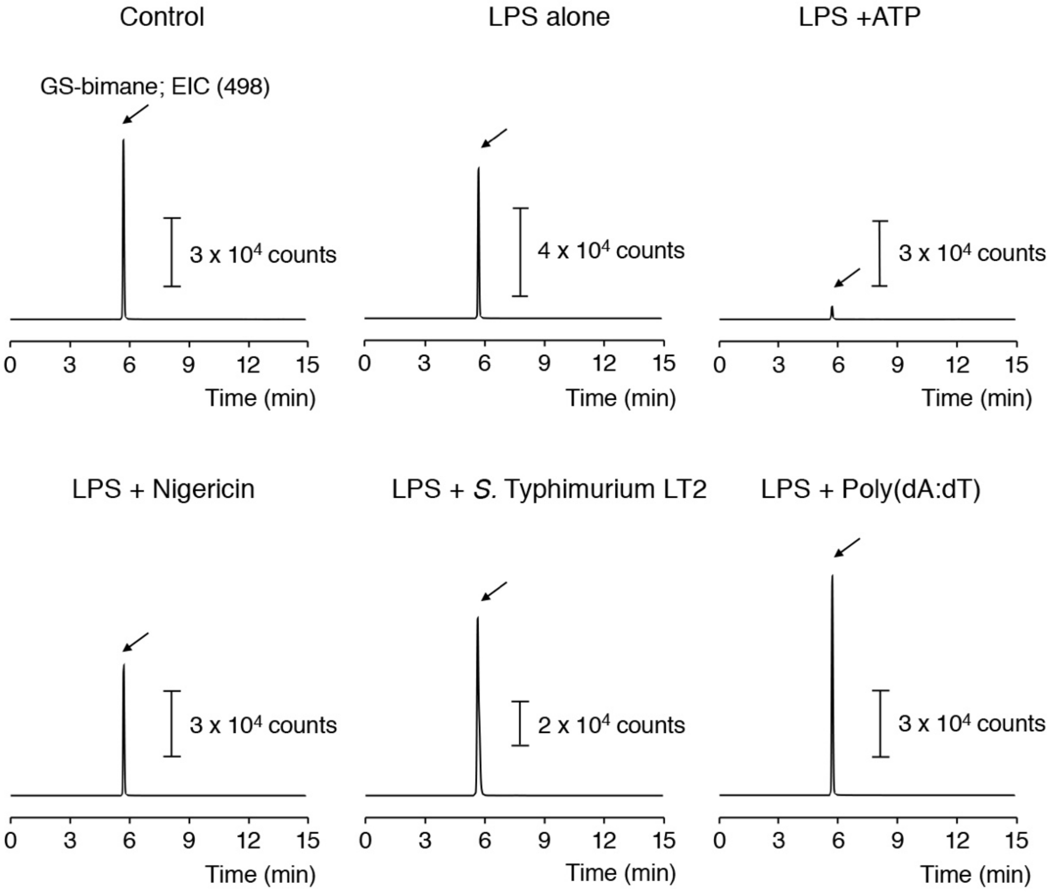

Figure S1. **EIC results for intracellular GSH levels of LPS-primed J774.1 cells in response to inflammasome activators.** Peaks with the corresponding mass number indicate intracellular GSH levels (arrows). Control means cells not treated with either LPS or activators.

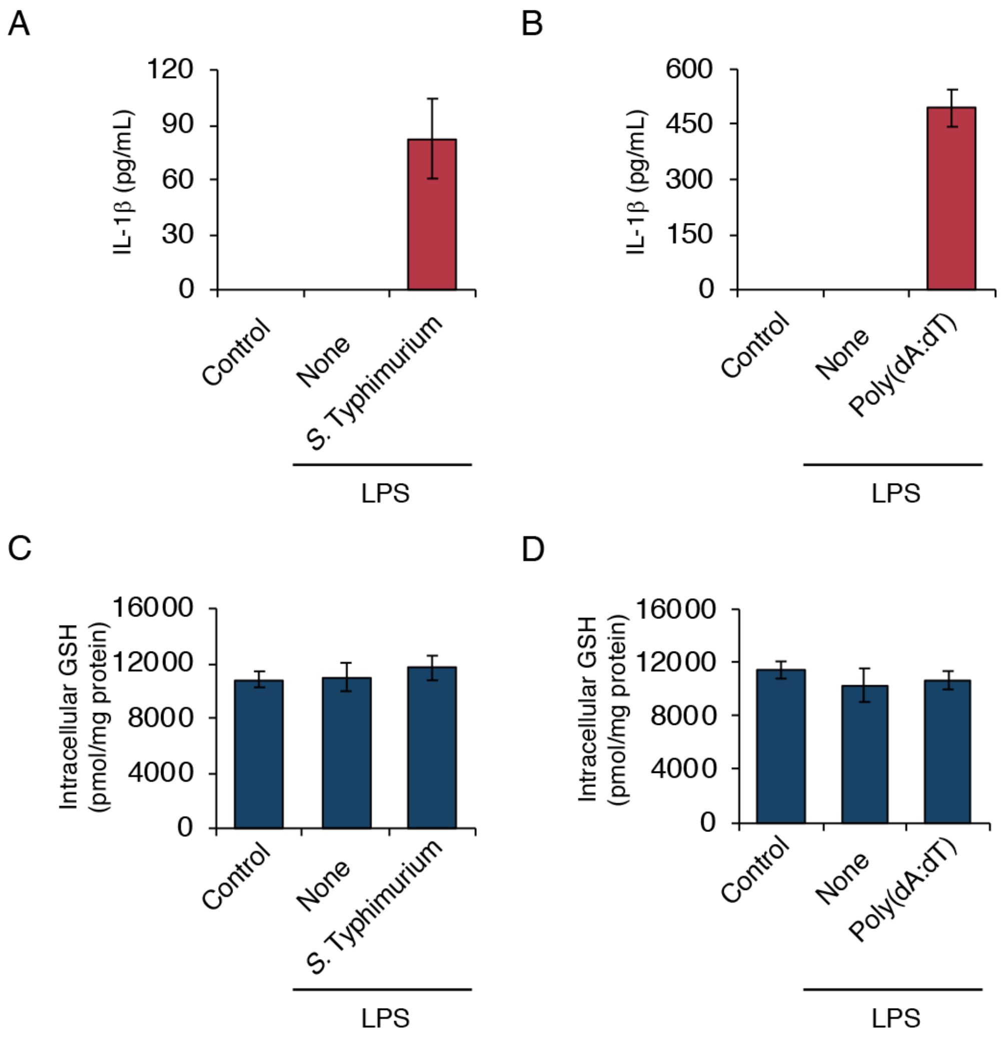

Figure S2. **Levels of intracellular GSH at the early stage of NLRC4 and AIM2 inflammasome activation.** J774.1 cells were primed with LPS (100 ng/mL) for 5 h, followed by infection with *S.* Typhimruim LT2 (multiplicity of infection = 1) or transfection with poly(dA:dT) (500 ng/mL). An hour later, (A and B) IL-1β levels in culture supernatants were determined by ELISA. (C and D) Intracellular GSH were measured by means of LC-MS/MS. Controls were cells not treated with LPS or stimulus. Data represent means ± SD (n = 3).

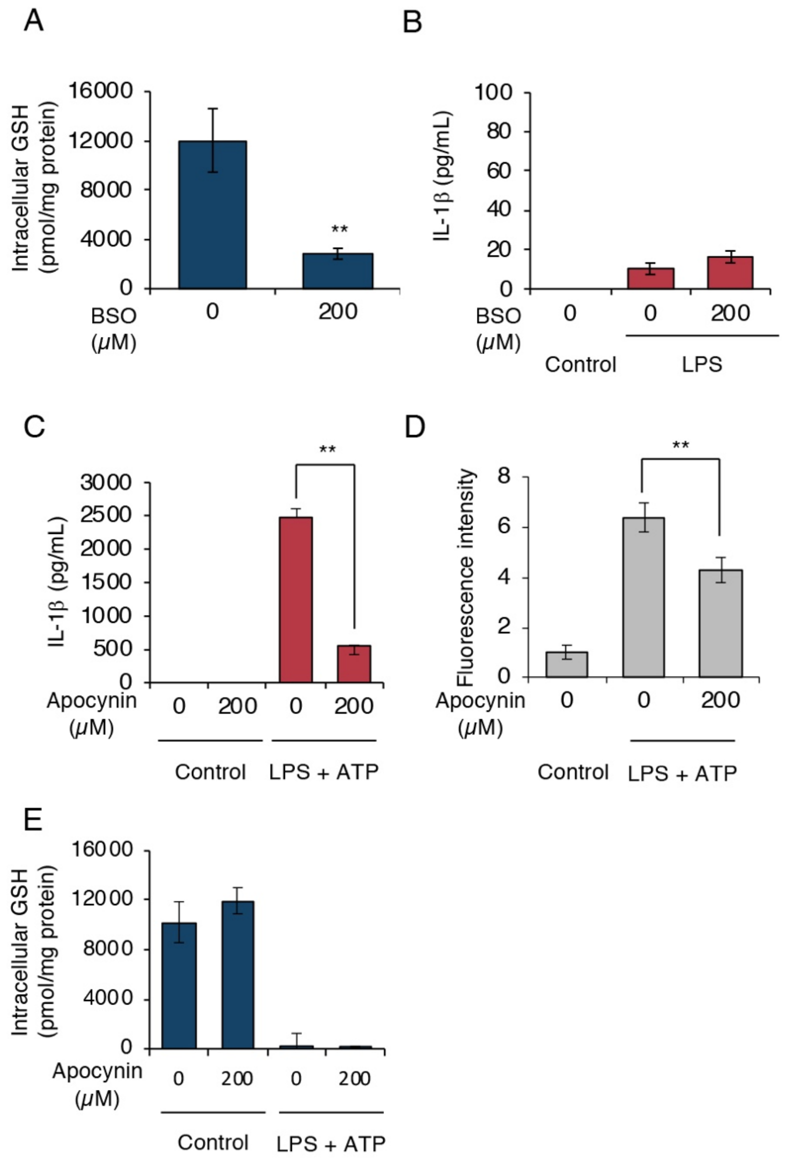

Figure S3. **ATP treatment activates the NLRP3 inflammasome in the presence of BSO and apocynin.** (A and B) Unprimed J774.1 cells were pretreated with BSO (200 μM) for 16 h. After incubation, LPS (100 ng/mL) was added to these cells and cells were cultured for 5 h, followed by stimulation with ATP (5 mM) for 1 h. (A) Intracellular GSH levels were confirmed via LC-MS/MS. (B) IL-1β release was quantified by using ELISA. (C-E) J774.1 cells were primed with LPS (100 ng/mL), or were not primed, in the absence or presence of apocynin (200 μM), after which they were stimulated with ATP (5 mM) for 1 h or were not so stimulated. (C) IL-1β production and (E) intracellular GSH levels were measured by using the same methods as those used in (A and B). (D) Quantitative data for the measurement of ROS generation in these cells. Controls were cells not treated with either LPS or ATP. Data represent means ± SD (n = 3). **p < 0.01.

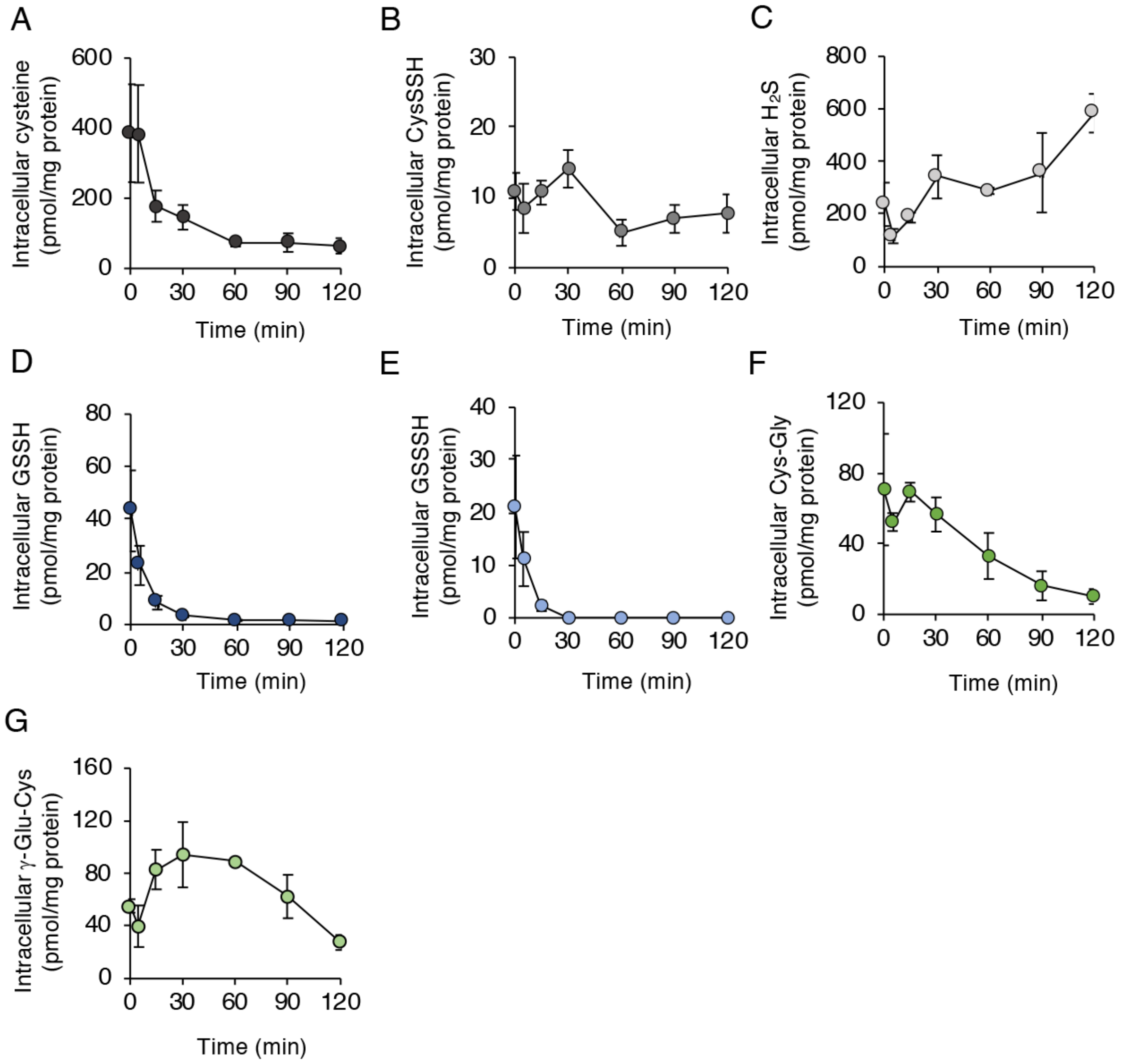

Figure S4. **LC-MS/MS analyses determine GSH metabolites.** J774.1 cells were primed with LPS (100 ng/mL) for 5 h and then stimulated with ATP (5 mM) for the indicated time periods. We used LC-MS/MS-based time-dependent GSH metabolomic analysis to quantify GSH derivatives including (A) cysteine, (B) CysSSH, (C) hydrogen sulfide (H_2S), (D) GSSH, (E) GSSSH, (F) Cys-Gly, and (G) γ-Glu-Cys. Controls were cells not treated with either LPS or ATP. Data represent means ± SD (n = 3).

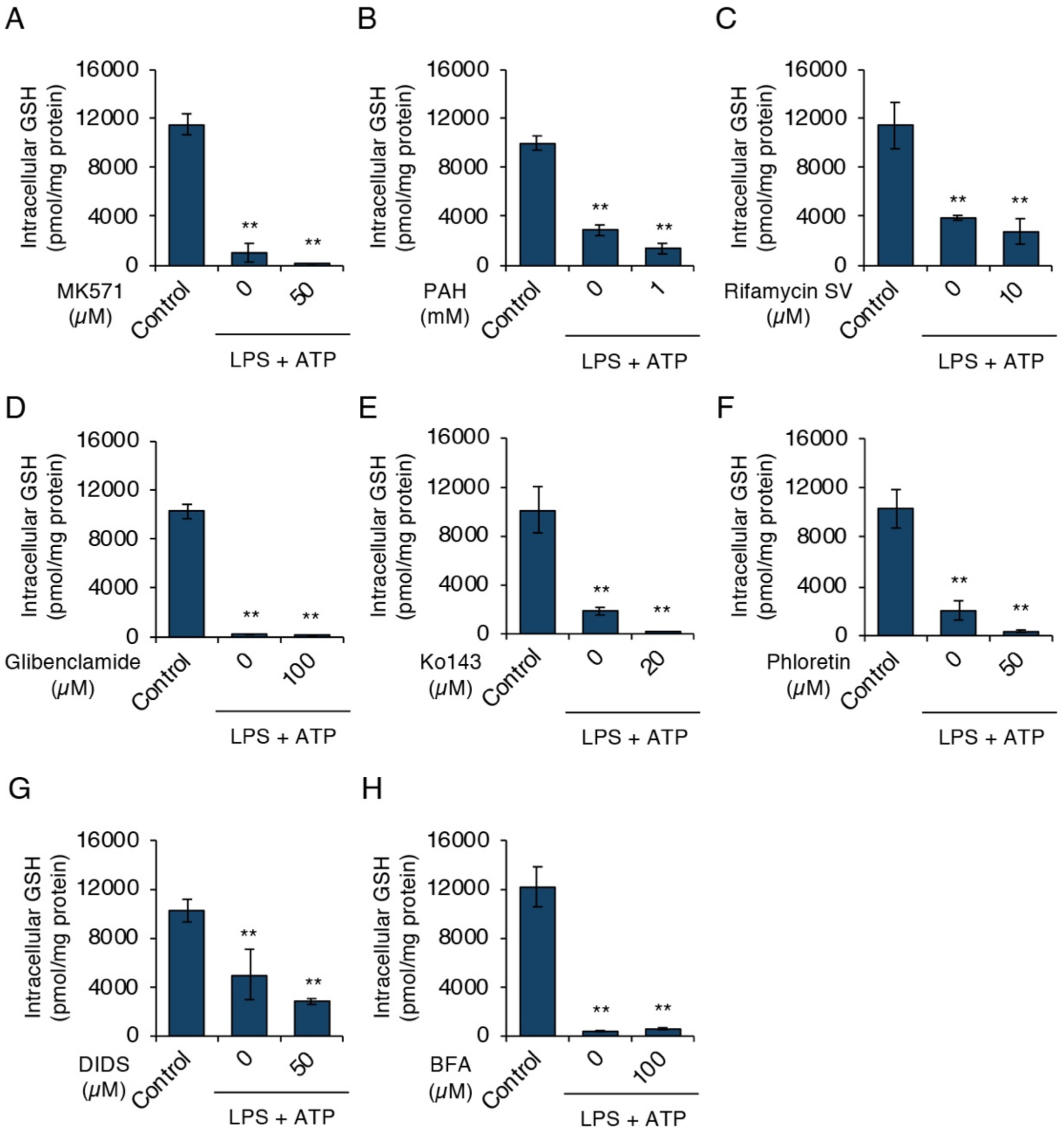

Figure S5. **GSH transporter inhibitors and channel blockers show no effect on GSH efflux after ATP treatment.** J774.1 cells were pretreated with (A) MK571 (inhibitor of multidrug resistance proteins), (B) PAH (OATs inhibitor), (C) rifamycin SV (inhibitor of OAT polypeptides), (D) glibenclamide (cystic fibrosis transmembrane conductance regulator blocker), (E) Ko143 (ABCG2 inhibitor), (F) phloretin (VRAC blocker), (G) DIDS (canalicular GSH transporter inhibitor), or (H) BFA (ER-Golgi protein trafficking inhibitor) at the indicated concentrations. After 1 h of incubation, LPS (100 ng/mL) was added to the cells, which were cultured for 5 h, followed by stimulation with ATP (5 mM) for 1 h. Intracellular levels of GSH were analyzed with LC-MS/MS. Controls were cells not treated with either LPS or ATP. Data represent means ± SD (n = 3). $^{**}p < 0.01$.

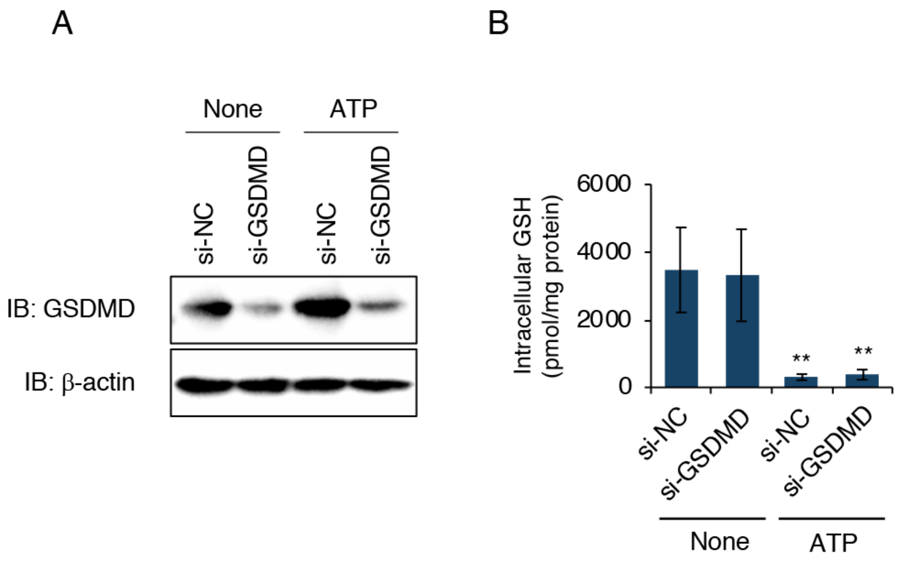

Figure S6. **Down-regulation of GSDMD in J774.1 cells shows no effect on GSH efflux after ATP exposure.** J774.1 cells were transfected with si-NC (100 nM) or si-GSDMD (100 nM) for 72 h. After washing once with DMEM, cells were treated with ATP (5 mM) for 1 h. (A) Knockdown of GSDMD in J774.1 cells was confirmed via Western blotting. (B) LC-MS/MS measurement for intracellular GSH levels in siRNAs-treated J774.1 cells after ATP exposure. Data represent means ± SD (n = 3). ** $p < 0.01$; si-NC., siRNA negative control; si-GSDMD., siRNA gasdermin D.

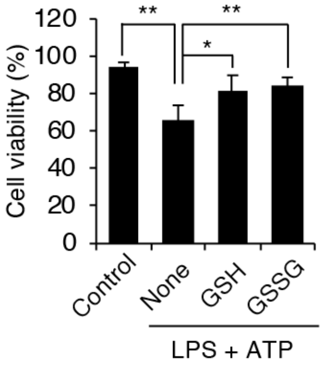

Figure S7. **Exogenous GSH and GSSG prevent cell death from ATP exposure.** J774.1 cells were primed with LPS (100 ng/mL) for 5 h, after which they were stimulated with ATP (5 mM) for 6 h in the absence or presence of GSH (5 mM) or GSSG (5 mM). Cytoprotective effects of GSH as well as GSSG were determined by trypan blue dye exclusion staining. Control were cells not treated with either LPS or stimulus. Data represent means± SD (n = 4). *$p < 0.05$; ** $p < 0.01$.

Table S1. **MRM Parameters for Monobromobimane Adducts and Related Molecules**

Analyte	Precursor Ion (*m/z*)	Product Ion (*m/z*)	Fragmentor Voltage (V)	Collision Energy (eV)	Polarity
[$^{13}C_2$,^{15}N]GSSG	619.2	361	170	21	+
GSSG	613.2	355	130	21	+
[$^{13}C_2$,^{15}N]GS-SS-bimane	565	192	170	41	+
GS-SS-bimane	562	192	170	41	+
[$^{13}C_2$,^{15}N]GS-S-bimane	533	192	130	37	+
GS-S-bimane	530	192	130	37	+
[$^{13}C_2$,^{15}N]GS-bimane	501	438	130	21	+
GS-bimane	498	435	130	21	+
GSH ethyl ester-bimane	526.2	463.1	130	21	+
γ-Glu-Cys-bimane	441.2	378	130	21	+
Bis-^{34}S-bimane ($H_2{}^{34}S$)	417.2	193	170	17	+
Bis-S-bimane (H_2S)	415.2	193	170	17	+
Cys-Gly-bimane	369.1	223	130	17	+
GSH ethyl ester	336.1	207.1	90	9	+
Cys^{34}S-SS-bimane	378	192	110	17	+
CysS-SS-bimane	376	192	110	17	+
Cys^{34}S-S-bimane	346	192	90	17	+
CysS-S-bimane	344	192	90	17	+
Cys^{34}S-bimane	314	227	130	17	+
CysS-bimane	312	225	130	17	+
H-Glu(Gly-Gly-OH)-OH	262.1	133.1	90	9	+
γ-Glu-Cys	251.1	84.2	90	29	+
Cys-Gly	179	76.2	50	13	+
